# New Understanding on the Pathophysiology and Treatment of Constipation in Parkinson’s Disease

**DOI:** 10.3389/fnagi.2022.917499

**Published:** 2022-06-22

**Authors:** Jianli Xu, Lei Wang, Xi Chen, Weidong Le

**Affiliations:** ^1^Institute of Neurology, Sichuan Provincial People’s Hospital, University of Electronic Science and Technology of China, Chengdu, China; ^2^Chinese Academy of Sciences Sichuan Translational Medicine Research Hospital, Chengdu, China

**Keywords:** Parkinson’s disease, constipation, molecular mechanism, non-pharmacological treatments, medication

## Abstract

Constipation, one of the most common prodromal non-motor symptoms of Parkinson’s disease (PD), usually occurs several years earlier than the onset of motor symptoms. Previous studies have shown that constipation occurrence increases as the disease progresses. However, the mechanism underlying this pathologic disorder is not clear yet. Moreover, chronic constipation causes slowness in gastric emptying and, therefore, may lead to a delay in the absorption of medications for PD, including levodopa and dopamine agonists. Accordingly, it is necessary to understand how the pathophysiological factors contribute to constipation during PD as well as pursue precise and effective treatment strategies. In this review, we encapsulate the molecular mechanism of constipation underlying PD and update the progress in the treatments of PD-associated constipation.

## Introduction

Parkinson’s disease (PD) is a complex chronic neurodegenerative disease highly prevalent with aging (Collaborators, 2019). Most PD cases are sporadic and less than 10% of them are familial. The prominent pathological feature of PD is the dopaminergic (DAergic) neuronal loss in the substantia nigra of the midbrain, leading to the significant reduction of DA content in the striatum and impairing the nigrostriatal projections (Carmichael et al., 2021). There are many contributing factors associated with the development of PD, including genetic and environmental factors (Samii et al., 2004; Simon-Sanchez et al., 2009). Currently, the diagnosis of PD mainly relies on its motor symptoms, such as resting tremor, bradykinesia, rigidity, and postural/gait abnormalities, based on the 2015 MDS clinical diagnostic criteria for PD (Postuma et al., 2015). However, these classical clinical features always occur at the advanced stage of disease progression. Recently, the non-motor symptoms of PD attract a special interest due to their early onset compared to motor symptoms and provide a promising perspective for early diagnosis and treatments of PD (Martinez-Martin et al., 2011; Bloem et al., 2021), including olfactory dysfunction, gastrointestinal (GI) dysfunction, mood symptoms, sleep disorders, autonomic dysfunction, and fatigue (Chaudhuri et al., 2006; Liu and Le, 2020). Constipation, a GI disturbance, is one of the most frequent non-motor symptoms and affects more than 80% of PD patients. Importantly, constipation may precede the motor symptoms of PD by at least 10 years (Fasano et al., 2015). Also, the colonic transit time is significantly prolonged in PD patients (Sakakibara et al., 2003; Knudsen et al., 2017; Zhang et al., 2021). The dysfunction of bowel movements undergoing PD may be contributed by many factors, such as neuro-humoral factors, intestinal microorganisms, intestinal inflammation, drugs, and lifestyle (Barichella et al., 2019). Currently, most of the therapeutic strategies for constipation applied in the general population are also effective for PD patients (Travagli et al., 2020). However, a better understanding of the pathophysiological mechanism underlying PD may promote the development of specialized treatments for constipation in PD patients. For example, deep brain stimulation (DBS) and vagal nerve stimulation (VNS), these two common surgical treatments for PD have been proven to mitigate constipation in PD patients as well, by facilitating intestinal emptying (Jost, 1997; Payne et al., 2019). In this review, we seek to encapsulate the molecular mechanisms discovered underlying constipation of PD, as well as update the progress of recent pharmacological and clinical findings.

### Pathophysiology of Constipation in Pd

**Figure 1:**
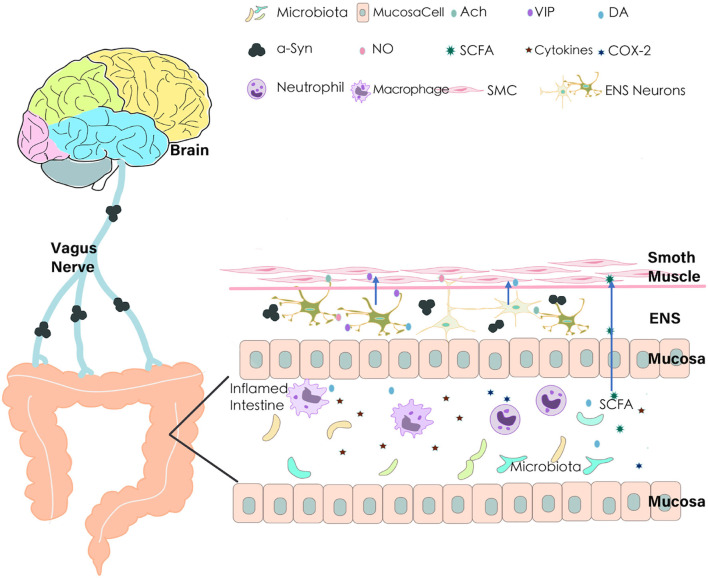
Pathophysiology of constipation in Parkinson’s disease (PD). Many factors may contribute to the alterations of colon transport and consequently lead to constipation in PD. Aberrant aggregation of α-Synuclein leads to the vagus nerve and ENS degeneration; dysfunction of the intestinal nervous system brings out the imbalance of secretion and regulation of neurotransmitters (NO, VIP, DA, Ach); gut microbiota imbalance and product of bacterial fermentation (such SCFAs) affect intestinal dysfunction; pro-inflammatory cytokines and COX-2 in the inflamed intestine leads to decrease of The gastrointestinal (GI) transport. ENS, Enteric Nervous System; VIP, Vasoactive Intestinal Peptide; NO, Nitric Oxide; Ach, Acetylcholine; DA, Dopamine; SCFA, Short-Chain Fatty Acids; COX-2, Cyclooxygenase-2; SMC, Smooth Muscle Cell; α-Syn, α-Synuclein.

Constipation in PD may occur due to the improper functioning of the autonomic nervous system, i.e., the intestinal tract may operate slowly and trigger constipation (Metta et al., 2022). Pathophysiology of constipation in PD is to discover the intrinsic and extrinsic factors contributing to PD-associated constipation (Warnecke et al., 2022). Generally, the normal function of the colon depends on the orderly and controllable unidirectional movement of its contents under the impetus of intestinal motility. Many factors have been known to affect colonic movements and intestinal contents, including neuro-humoral factors, gut microbiota imbalance, intestinal inflammation, drugs, and lifestyle. Once they are dysregulated, the transportability of intestinal contents is altered, resulting in constipation ultimately (as shown in Figure 1).

### Neuro-Humoral Factors

The innervation of the digestive tract determines its movement pattern, regulates the fluid movement of the intestinal cavity, and releases intestinal hormones (Furness et al., 2014). Different from other peripheral organs, the movement of the intestine is controlled by two distinct types of nerves. One is the extrinsic nervous system, which is composed of sympathetic and parasympathetic nerves. Sympathetic postganglionic fibers release norepinephrine, which can inhibit the excitation of neurons and further repress their forward conduction activities; the parasympathetic nerve, especially the vagus nerve, has a role in facilitating digestion-related enzyme, hormone secretion, and smooth muscle peristalsis. The other type of intestinal nerve system is the extensive intrinsic nervous system, the enteric nervous system (ENS). ENS consists of the intestinal wall intermuscular plexus and the submucosal nerve plexus, where a large number of sensory and motor neurons are located (Furness et al., 2014; Walsh and Zemper, 2019). The function of ENS is mainly in charge of contractile activity, local blood flow, and transmucosal movement of fluids (Furness, 2012). In PD, GI dysfunction is largely caused by the abnormalities of ENS and the vagus nerve (Quigley, 1996).

Aggregated α-Synuclein (α-Syn) is the major constituent of Lewy bodies, which are a pathogenic hallmark of PD (Li and Le, 2020). The vagus nerve is considered to be the main route of transmission of α-Syn. There is a degree of vagus nerve atrophy in the process of PD (Del Tredici and Braak, 2016), inhibiting gastric emptying and intestinal transport (Grijalva and Novin, 1990). Moreover, aggregate dα-Syn has also been found in the peripheral nerves including the ENS (Casini et al., 2021). The number of Lewy neurites in ENS is negatively correlated with neuron counts and positively correlated with constipation (Lebouvier et al., 2010). Therefore, aberrant accumulations of α-Synmay trigger vagus neurodegeneration, slow down intestinal peristalsis, and promote constipation (Liddle, 2018).

The dysfunction of the intestinal nervous system may bring out the imbalance of secretion, and dysregulation of intestinal neurotransmitters, further aggravating the disorder of intestinal motility. Vasoactive intestinal peptide (VIP), innervating the mucosa throughout the small and large intestines, plays a major role in the digestive system to relax intestinal smooth muscle and promote the secretion of intestinal fluid (Schwartz et al., 1974; Larsson et al., 1976). The studies from Giancola et al. (2017) showed that the impaired colonic motor and rectal sensory in most PD patients were associated with a decreased VIP expression in submucosal neurons. Such reduction of intestinal VIP potentially leads to intestinal diastolic disorder and loss of normal intestinal peristalsis mode, further limiting colonic transport and reducing intestinal fluid secretion. Secretory abnormalities may thereby affect the composition of the fecal water content, leading to hard stools and delayed colonic transit time (Lam et al., 2016).

Nitric oxide (NO), an inhibitory neurotransmitter secreted by NOS (nitric oxide synthase)—positive neurons in ENS, regulates the relaxation of smooth muscle, to maintain normal colon motility, colonic reflexes, and defecation (Stark et al., 1993). NOS is the key rate-limiting enzyme for NO production. In normal physiological conditions, NOS causes tissues to slowly release NO and maintains the body’s physiological requirements. When the body is in a pathological state, deficient NO production will affect intestinal transport (Shah et al., 2004). Studies with the animal models of PD confirmed that loss of NOS impairs the expression of the antioxidant gene, which deregulates NO synthesis and increases abnormal aggregation of α-Syn in ENS, thereby contributing to the development of GI dysmotility and constipation (Sampath et al., 2019). Additionally, NO appears to be important to maintain DA synthesis in the colon. The signals from NOS–NO may influence DA neurotransmission, and loss of NOS–NO signaling in the GI system may induce DAergic neuronal degeneration *via* oxidative stress, aggravating the degree of dyskinesia and constipation (Sampath et al., 2019).

Cholinergic neurons are the most abundant neurons in the ENS. The cells of cholinergic excitatory nerves are scattered under the intestinal mucosa and in the myenteric plexus. Acetylcholine, the neurotransmitter released by cholinergic neurons, stimulates the muscarinic receptors on smooth muscles and nicotinic receptors on ganglion cells, affects GI muscle excitatory and participates in intestinal peristalsis reflex (Picciotto et al., 2012). Therefore, alterations in excitatory cholinergic neurotransmitters have a significant impact on the movements of the distal colon. In PD, the loss of myenteric neuronal choline acetyltransferase and decreased acetylcholine release, likely contribute to the reduction of colonic transit rate by affecting the formation of excitatory electrical activity in the colon (Fornai et al., 2016), resulting in the impairment of fecal output and leading to constipation (Fidalgo et al., 2018).

DA is a key neurotransmitter for colon movements and is widely distributed to the intestine regions, including the colon muscle layer, intermuscular plexus, and mucosal epithelial cells. Therefore, dysfunction of DA receptors (DR) can affect GI transport (Li et al., 2006). It was reported that the rats with PD-related constipation show a significant reduction in D2R expression in the colon (Levandis et al., 2015). DA dysfunction is also closely related to DA transporter (DAT). DAT, a membrane protein, removes DA from the synaptic space, deposits it into surrounding cells, and terminates the signal of neurotransmitters. The neuroimaging data from PD patients showed that constipation is most closely associated with caudate-DAT reductions (Hinkle et al., 2018). Additionally, DA itself regulates GI transport as well. DA dysfunction weakens its inhibitory effect on gut motility and leads to spasms and hyper-contractility of colonic smooth muscles, which slows down intestinal transport (Anderson et al., 2007). In such a situation, an enteric DA deficit induces constipation in PD patients.

### Gut Microbiota Imbalance

The intestinal microbiota is a complex system composed of parasites, viruses, yeast, and bacteria (Gasbarrini et al., 2007; Jandhyala et al., 2015). The composition of the microbiota and the diverse metabolites they produce are widely related to the health of the host. Studies on gut microbiota have shown that PD patients are prone to show intestinal flora imbalance. A lower proportion of the bacterial phylum *Bacteroidetes* and the bacterial family *Prevotellaceae* were both identified in fecal samples from PD, where *Enterobacteriaceae* were more abundant instead (Unger et al., 2016). Such microbiota imbalance has been associated with motor dysfunction, GI injury, and colon transport time increase *via* affecting neurotransmitters and microbial metabolites (Scheperjans et al., 2015; Vandeputte et al., 2016; Cirstea et al., 2020; Shao et al., 2021). Short-chain fatty acids (SCFAs) are the main product of bacterial fermentation of dietary fiber or glycosylated host proteins such as mucins in the colon (Cummings et al., 1987; Park et al., 2019). SCFAs play an important role in acting as an energy source for colonocytes (Kaiko et al., 2016), regulating the gut barrier (Kelly et al., 2015), influencing inflammatory responses (Inan et al., 2000), enhancing neuronal survival, and promoting enteric neurogenesis (Vicentini et al., 2021). Thus it is essential to maintain SCFA homeostasis in the gut microbiota (Yang and Chiu, 2017; Cirstea et al., 2020). Moreover, the SCFA concentrations were significantly reduced in fecal samples of PD patients, potentially contributing to GI dysmotility (Unger et al., 2016).

### Intestinal Inflammation

Intestinal inflammation has been proposed to mediate gut-to-brain PD progression (Sharma et al., 2019). It was reported that the expression levels of pro-inflammatory cytokines and the markers of glial cells were significantly increased in the colon of PD patients (Devos et al., 2013), suggesting that GI inflammation is associated with PD. Furthermore, patients with PD-related constipation have shown the decreased content of SCFAs, the increased intestinal permeability, and the down-regulated regulatory T cells, all of which accelerate the neuronal inflammation in ENS (Hirayama and Ohno, 2021). Meanwhile, the induced inflammatory mediators promote the infiltration of neutrophils and macrophages into the smooth muscle layer and the production of NO, leading to the reduction of contractility of smooth muscle cells (Turler et al., 2006). Importantly, in the inflamed intestine, the increased expression of cyclooxygenase (COX)-2 resulted in a significant augment of prostaglandins within the circulation and peritoneal cavity, which aggravates the contractility dysfunction of GI smooth muscles and leads to constipation (Schwarz et al., 2001).

### Adverse Drug Reactions and Lifestyle

Constipation can be a side effect of PD drugs. A population-based study investigated that constipation was slightly increased after 1 year of DA treatments in PD patients, most of whom were using Levodopa (Pagano et al., 2015). Thus, Levodopa may exacerbate delayed gastric emptying and constipation by affecting DR to slow down intestinal movements (Bestetti et al., 2017). Moreover, ropinirole, bromocriptine, and piribedil can promote a high incidence rate of constipation, accompanied by nausea, dyskinesia, hallucination, dizziness, and somnolence symptoms (Li et al., 2017). On the other hand, at the advanced stage of disease progression, PD patients move slowly with limited activity, and even stay in bed for a long time, which may lead to slow GI peristalsis and constipation. Dysphagia is commonly seen in older adults with PD, who usually experience dehydration (Thiyagalingam et al., 2021). Such dehydration may induce the release of antidiuretic hormone and aldosterone to increase the salt and water absorption in the colon, resulting in constipation in PD (Read et al., 1995).

### Treatments

**Figure 2:**
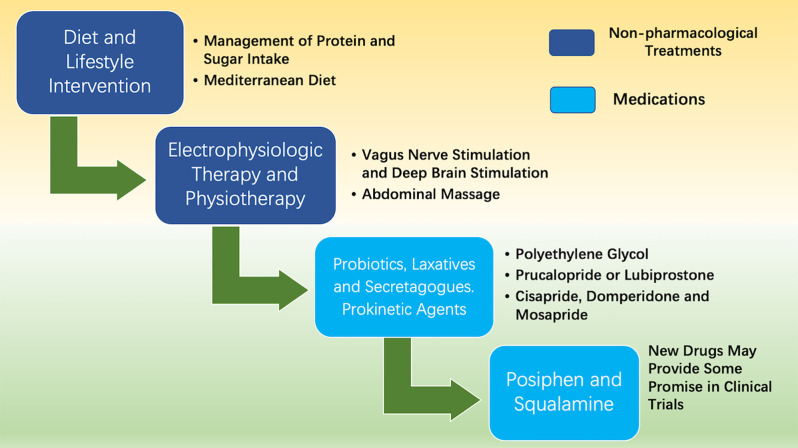
Treatments of constipation in PD. The treatments for PD patients with complicated constipation include diet control, electrophysiologic therapy, physiotherapy, and medications.

Currently, the treatments for idiopathic chronic constipation are equally effective in treating PD-associated constipation, including lifestyle alteration, diet control, and medication (Black and Ford, 2018; as shown in Figure 2). In recent years, DBS as an effective surgical procedure for PD, not only improves motor dysfunction but also alleviates constipation symptoms in PD patients (Hogg et al., 2017; Kahan et al., 2019). Thus, the development of drug and therapeutic strategies for PD will greatly benefit the treatments of constipation in PD patients.

## Non-pharmacological Treatments

### Diet and Lifestyle

Diet and lifestyle interventions play an important role in managing constipation (Fathallah et al., 2017). A large-scale lifestyle study found that PD patients consumed more energy and protein, compared to controls. It was known that protein can stimulate insulin and incretin hormone secretion as well as slow gastric emptying (Ma et al., 2009). Thus, the increased protein intake may reduce oral levodopa absorption and aggravate constipation symptoms (Fasano et al., 2015; Barichella et al., 2017). Additionally, another diet survey demonstrated that the sugar intake of PD patients increased significantly, accompanied by aggravated severity of constipation and a greater need for levodopa (Palavra et al., 2021). Thus, the management of protein and sugar intake should be considered an integral part of the care of PD patients. Furthermore, after providing a vegetarian diet and bowel cleansing for PD patients, UPDRS III (Unified Parkinson Disease Ratings Scale III) can be significantly improved, and levodopa usage was decreased (Hegelmaier et al., 2020). UPDRS III is an internationally well-established rating scale for assessing the motor symptoms of PD patients. Additionally, the Mediterranean diet is considered a combination of healthy foods and has been proved to be negatively correlated with multiple prodromal features of PD, including constipation. The studies indicated that the higher score for adherence to the Mediterranean diet, the more obvious probability of constipation relief. Thus, a healthy diet pattern can improve the constipation symptom of PD patients at the early stage (Maraki et al., 2019; Molsberry et al., 2020). Together, these findings suggest that dietary management may help to maintain nutritional status, optimize levodopa treatment, and minimize its related motor complications.

### Surgical Treatment

The GI tract has substantial two-way neural interactions with the central nervous system through the vagus nerve (Powell et al., 2017). Thus, the vagus nerve, the channel of information exchange, is an attractive target of neurostimulation therapy for the treatment of GI disorders (Breit et al., 2018). Utilization of bioelectronic therapy has been applied to the vagus nerve, called VNS. VNS activated the dorsal motor nucleus (DMV), reduced intestinal pro-inflammation cytokine expression, decreased leukocyte recruitment to the manipulated intestine segment, and eventually improved GI transit (Hong et al., 2019). Importantly, VNS is well tolerated with fewer relevant side effects (such as abdominal pain, flatulence, and bloating), and thereby is considered to be a promising non-invasive therapy to improve gastroenteric symptoms in PD (Kaut et al., 2019).

DBS is a surgical technique in which one or more electrodes are attached to leads and implanted in specific regions of the brain (Malek, 2019). Two specific sites in the brain have been the most common targets for DBS in PD: the subthalamic nucleus and the internal segment of the globus pallidus (Morishita et al., 2013). A follow-up evaluation found that DBS effectively ameliorated motor symptoms, and greatly adapted non-motor symptoms, including constipation (Zibetti et al., 2007). After DBS, the severity of constipation was significantly improved and the number of complete spontaneous bowel movements remarkably increased (Kola et al., 2021). Animal experiments also indicated that DBS in rats could accelerate colonic transit and increase colonic motor activity through the central DAergic pathway (Derrey et al., 2011).

Additionally, abdominal massage is perceived to be beneficial in relieving symptoms of constipation for PD patients (McClurg et al., 2016b). It can stimulate peristalsis, reduce colonic transit time, increase the frequency of bowel movements in constipated patients, and also alleviate the feelings of discomfort and pain that accompany constipation (Sinclair, 2011). Some experts believe that abdominal massage, an adjunct to the management of constipation, offers an acceptable and potentially beneficial intervention for PD patients (McClurg et al., 2016a).

### Medications

Chronic constipation is the most frequent symptom of autonomic system involvement in PD, which becomes severe as the disease progress and greatly impairs the life quality of patients (Stocchi and Torti, 2017). Considering the efficacy and cost, management of constipation should begin with diet or laxatives. Laxatives are commonly used to treat constipation. Although initial observations suggested that long-term use of laxatives could induce ENS damage, more evidence and guidelines indicate that stimulant laxatives, such as polyethylene glycol, are safe and effective for chronic constipation (Siegel and Di Palma, 2005; Pare and Fedorak, 2014).

If patients are not responding to traditional laxatives, intestinal secretagogues or prokinetic agents can also be accounted for in the constipation treatment, such as prucalopride or lubiprostone (Black and Ford, 2018; Bharucha and Lacy, 2020). Prucalopride is a selective agonist of serotonin 5-HT_4_ receptors. With a favorable safety profile, prucalopride is effective in many forms of constipation, such as opioid-induced constipation usually appearing at the early stages of PD (Omer and Quigley, 2017). Lubiprostone, working by increasing fluid and electrolyte flux into the intestinal lumen, seemed to be well tolerated and effective for the short-term treatment of constipation in PD (Ondo et al., 2012). Cisapride, a prokinetic agent, can enhance the physiological release of acetylcholine from postganglionic nerve endings of the myenteric plexus and stimulate bowel movement (Neira et al., 1995). In addition, domperidone and mosapride can also stimulate digestive gastrointestinal tract motility, thereby ameliorating constipation in PD patients (Soykan et al., 1997; Liu et al., 2005).

Recently, several new drugs have been developed and are considered to be potential for the treatment of constipation in PD. Posiphen, an α-Syn protein translation inhibitor, was confirmed to normalize the colonic motility in mouse models with GI dysfunction (Kuo et al., 2019). Further study in humans showed that posiphen is well tolerated and significantly lowers inflammatory markers (Maccecchini et al., 2012). Squalamine, a zwitterionic amphipathic amino sterol, originally isolated from the liver of the dogfish shark, dramatically affects α-Syn aggregation *in vitro* and *in vivo* (Perni et al., 2017). After taking squalamine in the PD mouse model, the excitability of intrinsic primary afferent neurons of ENS can be rapidly restored (West et al., 2020). A double-blind, placebo-controlled study also confirmed that oral tablets of squalamine are safe and significantly improve bowel function in PD-related constipation (Hauser et al., 2019). Together, these drugs may provide some promise in clinical trials but need to be evaluated further.

Besides chemicals, multi-strain probiotics are also considered an effective strategy for alleviating constipation, by restoring the balance of gut microbiota, increasing the intestinal opening frequency, and improving the whole intestinal transmission time (Westfall et al., 2017; Tan et al., 2021). The consumption of fermented milk containing multiple probiotic strains and prebiotic fiber was superior to placebo in improving constipation in patients with PD (Barichella et al., 2016). Such treatments can greatly improve the quality of life in PD patients with constipation (Ibrahim et al., 2020; Tan et al., 2021).

### Summary and Prospects

Since the relationship between the pathogenesis of constipation and disease progression in PD patients is still unclear, no completely effective radical therapeutic method is identified yet. Currently, the clinical treatments of constipation in PD patients are similar to the treatments in the general population. Although these managements have limited effects in the short term, the long-term efficacy is still poor. Thus, more efforts in the studies of PD pathogenesis may be needed to expand the scope of research, provide new strategies, and improve the life quality of patients.

## Author Contributions

LW conceptualized the manuscript. JX and WL drafted the manuscript. LW and XC revised and supervised the manuscript. All authors contributed to the article and approved the submitted version.

## Funding

This work was supported by funding from the Key Project of the Medical Science Department, University of Electronic Science and Technology of China (ZYGX2020ZB035 to LW), Guangdong Provincial Key R&D Program (2018B030337001 to LW), the Youth Program of National Natural Science Foundation of China (81901405 to XC), the Key Research and Development Program of Sichuan (2021YFS0382 to XC).

## Conflict of Interest

The authors declare that the research was conducted in the absence of any commercial or financial relationships that could be construed as a potential conflict of interest.

## Publisher’s Note

All claims expressed in this article are solely those of the authors and do not necessarily represent those of their affiliated organizations, or those of the publisher, the editors and the reviewers. Any product that may be evaluated in this article, or claim that may be made by its manufacturer, is not guaranteed or endorsed by the publisher.
